# Model Study of the Pressure Build-Up during Subcutaneous Injection

**DOI:** 10.1371/journal.pone.0104054

**Published:** 2014-08-14

**Authors:** Maria Thomsen, Anier Hernandez-Garcia, Joachim Mathiesen, Mette Poulsen, Dan N. Sørensen, Lise Tarnow, Robert Feidenhans'l

**Affiliations:** 1 Niels Bohr Institute, University of Copenhagen, Copenhagen, Denmark; 2 Novo Nordisk A/S, Hillerød, Denmark; 3 Nordsjæ llands Hospital, Hillerød, Denmark; 4 Klinisk Epidemiologisk Afdeling, Aarhus Universitetshospital, Aarhus, Denmark; 5 Steno Diabetes Center A/S, Gentofte, Denmark; University of California San Diego, United States of America

## Abstract

In this study we estimate the subcutaneous tissue counter pressure during drug infusion from a series of injections of insulin in type 2 diabetic patients using a non-invasive method. We construct a model for the pressure evolution in subcutaneous tissue based on mass continuity and the flow laws of a porous medium. For equivalent injection forces we measure the change in the infusion rate between injections in air at atmospheric pressure and in tissue. From a best fit with our model, we then determine the flow permeability as well as the bulk modulus of the tissue, estimated to be of the order 10^−11^–10^−10^ m^2^ and 10^5^ Pa, respectively. The permeability is in good agreement with reported values for adipose porcine tissue. We suggest our model as a general way to estimate the pressure build-up in tissue during subcutaneous injection.

## Introduction

Diabetics are treated by several daily injections of insulin, most commonly delivered subcutaneously from where the insulin gets absorbed by the blood vessels. The injections are typically performed using an insulin pen or normal syringe. Due to the high frequency of injections for diabetics, increased patient convenience is of great importance and devices are therefore continuously improved to simplify treatment.

The force delivered by injection devices, either injection pumps or auto-injection devices, must overcome both the resistance in the injection system and the resistance in the body tissue to make room for the insulin bolus. The second part we refer to as the tissue counter pressure. The subcutaneous compartment is decomposed mainly of two components; adipose tissue and interstitial tissue. Adipose tissue consists of adipocytes assembled in lobules separated by a thin layer of connective tissue and nerves and blood vessels running between the lobules. The interstitial tissue is placed between the adipocytes and consists mainly of a fibre framework made of collagen embedded in a mucopolysaccharide gel [1], [2]. Insulin therapy may lead to skin disorders, where the most common is lipohypertrophy, where extra adipose tissue is accumulated under the skin. The risk of developing lipohypertrophy increases with the time the patients have been treated, the frequency of changing the needle and injection site [3]–[6]. Lipoatrophy is a rarer condition, mostly related to impurities in insulin formulations. In lipoatrophy the size of the adipocytes in subcutis decreases around the injection site [7]–[9]. These kind of skin disorders impair in some cases the insulin absorption [5], [10], [11], but as the skin structure is changed it might also change the tissue counter pressure and affect the way the drug is delivered by the injection device.

The interstitial fluid pressure in subcutis has been measured by the wick clamp technique to be of the order −1.3 mbar, when not subjected to mechanical stress [13], [14] and it has been shown that the tissue counter pressure in subcutis increases with increasing infusion rate for continuous saline infusion from about 8 mbar to 60 mbar for infusion rates from 0.16* µ*L/s to 8.3* µ*L/s [15], in a study targeting insulin infusion pumps. To our knowledge the infusion pressure has not been measured for subcutaneous injections at injection speeds of the order 100* µ*L/s, which is the common infusion rate using an insulin pen [16].

In this study we present a non-invasive technique for measuring the tissue counter pressure during a normal insulin injection. We use an auto-injection device, where a click signal gives information on the flow rate. By comparing the infusion rate for subcutaneous injections and reference injections in air, we calculate the tissue counter pressure. We propose a model for the pressure evolution in subcutaneous tissue and from that we estimate the flow permeability and bulk modulus of the tissue.

## Materials and Methods

### Ethical approval

The study involving human subjects was conducted in collaboration with Steno Diabetes Center A/S. While a nurse administered insulin to the patient, the audible clicks, emitted from the injection device, were recorded by microphone. Prior to the investigation The Committee on Health Research Ethics from the Capital Region of Denmark reviewed the project. The conclusion from the committee was that the project could be conducted without further ethical approval because of its limited intervention. The participating patients all had type 2 diabetes and received regular control of their diabetes at Steno Diabetes Center. The responsible nurse contacted the patients prior to their control visit and questioned them about participation in the study. The head of the Clinical Research Department, who was responsible for the conduct of the study, approved the inclusion of patients without written informed consent, after acceptance of this procedure by The Committee on Health Research Ethics from the Capital Region of Denmark. No institutional review process was necessary to take this decision. The only restriction to the selection of subjects was that their insulin dose should be above 18 units to have a sufficiently long sound file for the subsequent analysis. All patients that were asked to participate gave their consent.

### Experimental design

**Figure 1:**
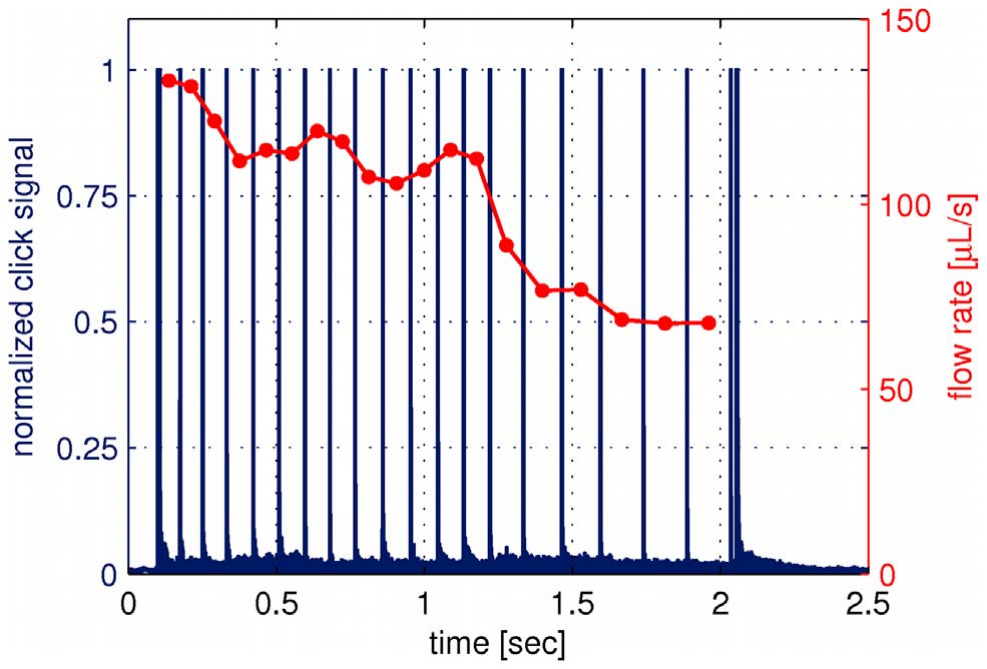
Click signal as emitted from the device and the flow rate. The click signal (blue curve) is measured by a microphone and the flow rate (red curve) is calculated from the time steps between the click signal. This example is for an injection of 240 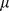
L in air.

To measure the pressure build-up in human tissue, we use the insulin pen FlexTouch from Novo Nordisk A/S, which is an auto injecting device, where the drug is injected by the force from a torsion spring. The spring is twisted when the patient dials the dose and as the spring is untwisted during the injection the device emits a click for each 10* µ*L of drug which has been delivered. By recording the click signal with a microphone we predict the flow rate. One example of a click signal and the calculated flow rate is shown in Figure 1 for an injection of 240* µ*L of insulin in air. It is seen that the flow rate decreases throughout the injection as the spring is untwisted and due to a special feature inside the pen the spring force is reduced for the last part of the click signal, seen as a drop in the flow rate after about 1.2 s. Therefore the last part of the click signal has been omitted in our analysis.

**Figure 2:**
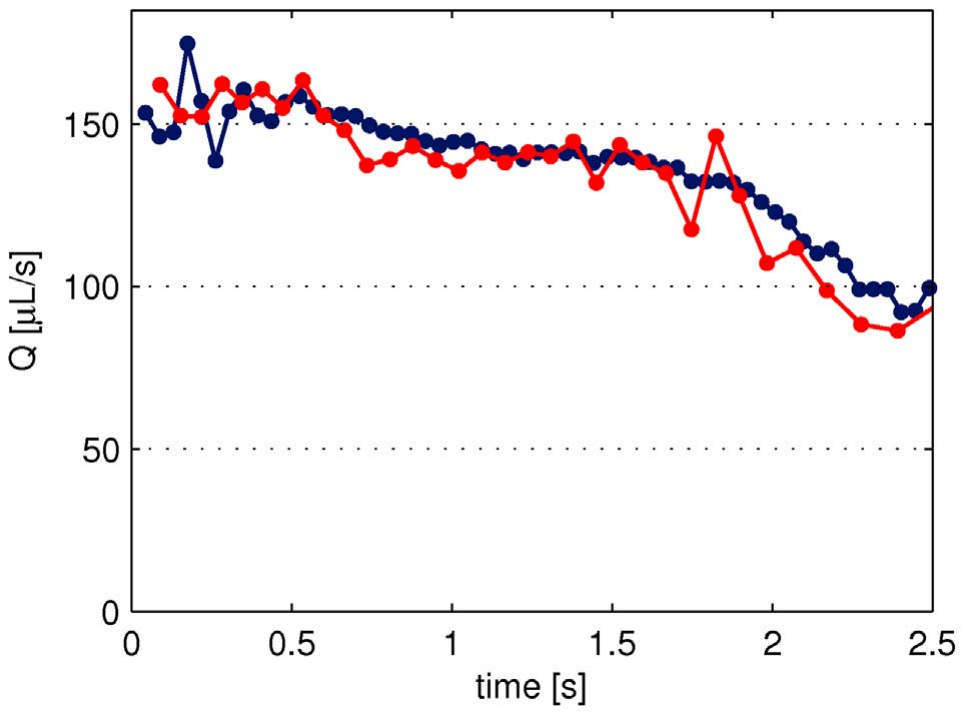
Comparison of flow rate calculated from the click signal and measured on a scale. The click signal and the injected mass are measured simultaneously, to give the flow rate calculated from the time steps between the click signals (red curve) and from the injected mass between equal time steps (blue curve). The injected volume was 400* µ*L.

In order to confirm that the flow rate calculated from the click signal match the actual flow rate a series of injections were performed in air where the click signal was recorded by a microphone and the injected mass was measured on a scale, simultaneously. Figure 2 shows an example of the flow rate as calculated from the click signal (red curve) and from the injected mass (blue curve). In total the flow rate was measured for 70 injections, using 10 different pens, and the injected dose was varied from 150–800* µ*L. On average the deviation between the flow calculated from the click signal and the injected mass was about 2%. This uncertainty is related to the flow rate prediction and not to the total dose delivered, which has been shown to be very accurate for this device [17], [18].

### Data collection

This study includes 11 men with type 2 diabetes. All the injections were given subcutaneously in the abdomen using a 6 mm 31G needle and performed by the same nurse. The patients were treated with NovoRapid U100 from Novo Nordisk A/S and the dose injected was set by the treatment scheme of the individual. Therefore the dose varies from 180* µ*L up to 480* µ*L. Both before and after the subcutaneous injection given to the patient, we record the click signal from a series of similar injections in air. The difference in the flow rate between the injections in air and in tissue relates to the counter pressure. After the injections the radius of each of the needles was calculated from Eq. (4) (see the Results section), by measuring the flow rate through the needle for a known pressure.

## Results

**Figure 3:**
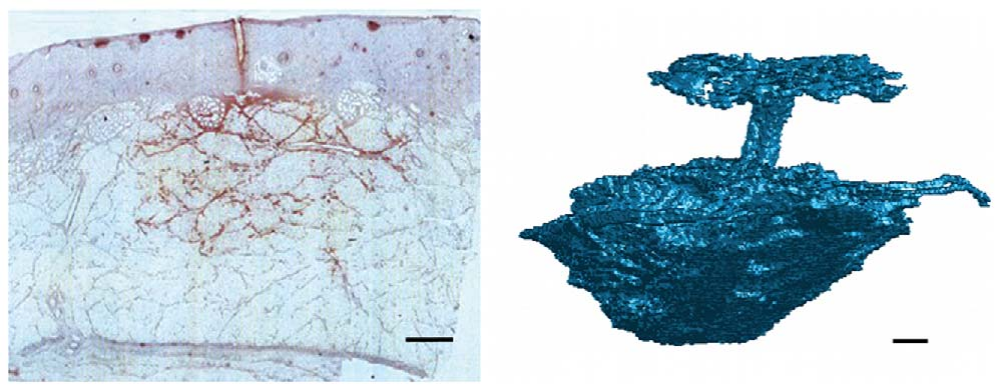
Histological cross section (left) and X-ray computed tomography scan (right) of similar subcutaneous injections. The injections were performed in adipose pig tissue and the injected volume was 100* µ*L. For histology the insulin has been dyed to appear red in the light microscopy image. The segmented tomographic reconstruction shows the 3 dimensional extension of the injection depot together with the injection channel and the backflow at the skin surface. The contrast between the tissue and the injected fluid is obtained by mixing the insulin drug with an iodine based contrast agent. The scale bar is 1 mm.

Insulin injected subcutaneously distributes between the fat lobulus and forms a depot, as seen from the histological cross section of a 100* µ*L insulin injection shown in Figure 3 (left). The insulin has been dyed to appear red and it is seen how the needle has penetrated dermis (blue skin layer). For the model of the tissue mechanics we consider the tissue as a porous elastic medium. Figure 3 (right) shows the 3 dimensional structure of a similar injection depot visualized by X-ray computed tomography, where the insulin drug has been diluted with an iodine based contrast agent in order to distinguish the fluid from the tissue, as described in a previous study [19]. The extension of the depot is about 1 cm and we have observed that the shape of the depot vary from injection to injection. For simplicity we assume, that the tissue is homogeneous and that the drug is distributed spherical symmetrically around the injection site.

In the description of the model we introduce a number of variables and constants, which we for clarity have listed in Table 1.

**Table 1:** Table of constants and variables introduced in the text.

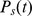	pressure at the inlet of the needle attached to the syringe [Pa]
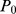	pressure at the outlet of the needle [Pa]
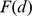	force delivered by the torsion spring [N]
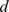	position of the piston head [m]
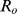	inner radius of the syringe [m]
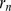	inner radius of the needle [m]
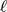	length of the needle [m]
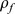	mass density of infused fluid in the tissue [kg/m^3^]
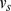	average velocity over the cross section of the needle inlet [m/s]
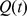	total injection rate in the tissue [m^3^/s]
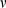	kinematic viscosity of the drug being infused [m^2^/s]
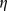	dynamic viscosity of the drug being infused [Pa⋅s]
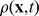	density of infused fluid in the tissue [kg/m^3^]
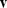	average velocity of the injected fluid in a unit volume of pore space [m/s]
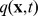	local injection rate of mass [kg/(m^3^⋅s)]
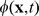	local volume fraction of infused fluid in the tissue [-]
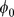	porosity of the tissue [-]
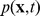	pressure in the tissue [Pa]
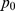	tissue pressure without distortion [Pa]
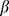	bulk modulus of the tissue [Pa]
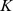	permeability of the tissue [m^2^]

### Mechanics of the injection device

**Figure 4:**
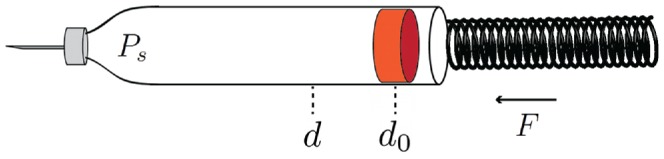
Schematic illustration of the insulin injection device. The force, *F*, acting on the piston from the torsion spring is a function of position, *d*, of the piston.

The mechanical characteristics of the injection device is analysed through a series of test experiments where the drug is injected in air at atmospheric pressure. A simple schematic illustration of the injection device is shown in Figure 4.

The pressure drop in the syringe due to viscous forces 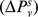 is vanishingly small when compared with the pressure drop in the needle 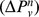. The pressure drop in the needle consists of a transient part, the entry length, over which the flow evolves to a steady state parabolic Poiseuille flow and a final part where the flow attains its parabolic shape (see Supporting Information S1). In the syringe the flow does not reach the full parabolic form during the short injections times considered here, however, an upper estimate of the pressure drop is achieved by assuming that the flow has reached a fully developed Poiseuille flow, which leads to 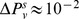
Pa. The pressure drop in the needle is estimated from the following expression derived in the Supporting Information S1, Eq. (S30),
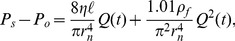
(1)where 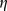 is the dynamic viscosity of the insulin drug, 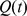 is the injection flow rate, 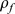 is the mass density of the drug and 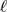 and 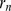 denote the length and radius of the needle, respectively. We get for typical injection flow rates *Q*(*t*)≈100* µ*L/s a pressure drop in the needle 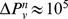 Pa. The pressure drop in the needle is therefore of the order 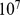 times larger than that in the syringe. Therefore, we shall neglect the viscous forces inside the syringe in our calculations below.

From the Bernoulli Equation we obtain that the pressure at the inlet of the needle is approximately given by
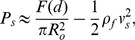
(2)where 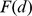 is the force delivered by the spring, 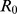 is the radius of the syringe and 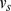 denotes the average velocity over the cross section at the needle inlet, which can be determined as



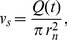
(3)Inserting Eq. (2) and (3) in Eq. (1), we obtain
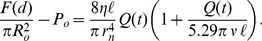
(4)


**Figure 5:**
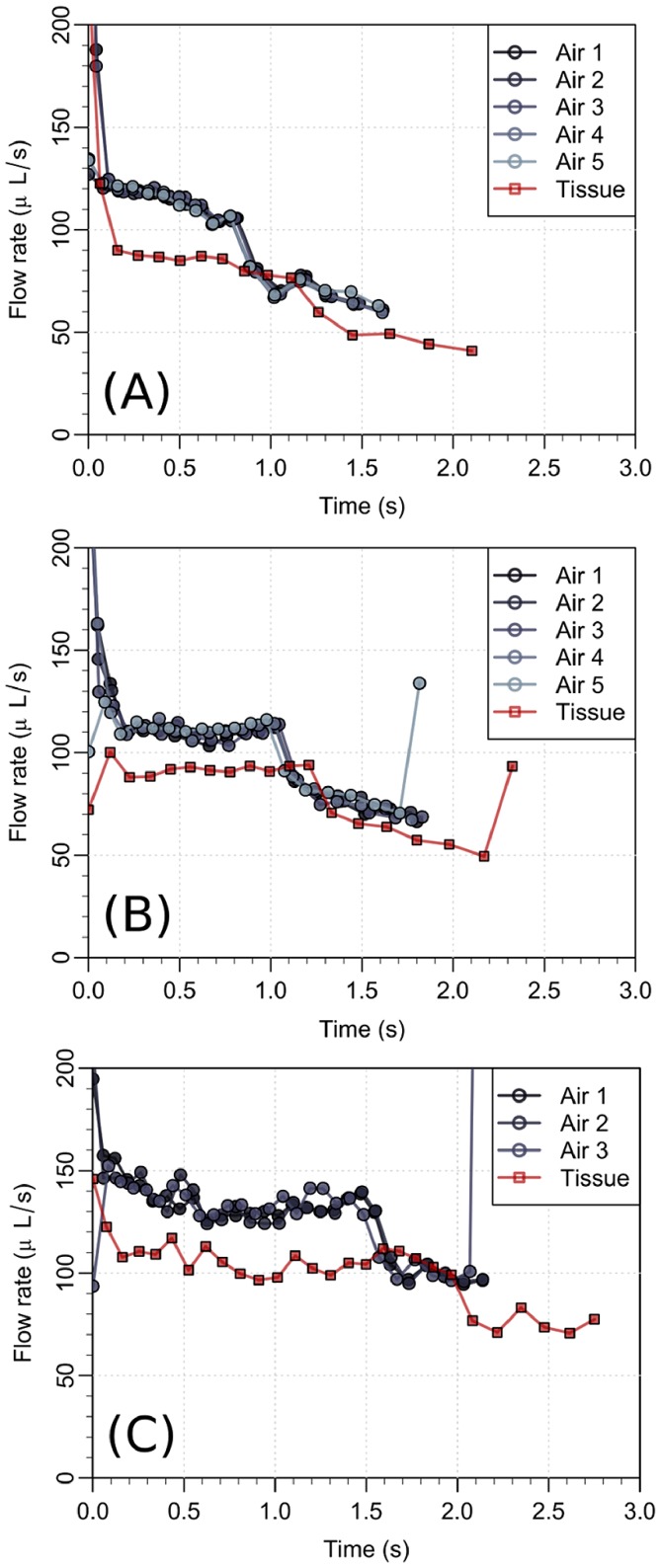
Flow rate during injection in air and in subcutis on three different patients. A significant drop in the flow rate is seen when the drug is injected in the tissue. The final and sudden jump in the flow rate in the panels (B) and (C) is due to an intended change in the mechanics of the syringe at end of the injection.



We now estimate the typical values of the second term inside the parentheses in Eq. (4). In Figure 5, we observe that the largest flow rates during the injection in air are approximately 150* µ*L/s. Using the value of the kinematic viscosity of water 
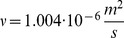
 and that the length of the needle is 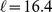 mm, we have that

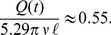
(5)As for the case of the fluid injection in the tissue, we also observe in Figure 5 that the largest flow rates are approximately 100* µ*L/s, which leads to
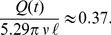
(6)


The second term in Eq. (4) therefore contributes significantly to the the pressure drop between the syringe and the tissue/air and is included in our model.

### Pressure in the tissue

In the derivation of an equation for the pressure evolution in tissue during injection we start from mass-conservation,
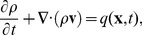
(7)where 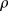 is the mass of the injected drug per unit volume 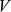, 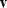 is the pore flow velocity in a unit volume and the local source term 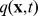 is the mass of drug being injected per volume per time. A unit volume in the tissue is assumed to comprises of the tissue (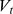) and the injected drug (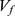), i.e.
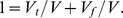



The drug density in a unit volume of tissue can be written in terms of the mass density of the drug (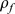) as 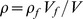, where 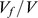 is the local volume fraction of the drug. We introduce a field for the local volume fraction of injected fluid 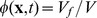 in Eq. (7) and end up with the equation:
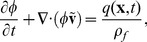
(8)where 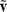 is the pore averaged flow velocity of the injected fluid in a unit volume. Below we shall only consider the averaged velocity and therefore for convenience omit the tilde over 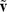.

The flow velocity is assumed to be given by the pressure gradient through Darcy's Law [12]

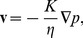
(9)in which 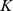 is the permeability, 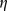 the dynamic viscosity of the fluid and 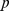 is the pressure.

Moreover we assume that displacement of the tissue by the injected fluid gives rise to a counter pressure, which will act to restore the tissue structure. This pressure is in the most simple approximation and for small deformations assumed to be linearly proportional to the difference in local tissue porosity before (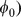 and after (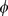) displacement by the injected fluid
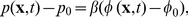
(10)where 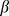 is an effective bulk modulus describing the force needed to locally displace the tissue. Note that this form of the pressure-porosity relation is only valid for relatively small pressures, since for a very large pressure this relation might even lead to a porosity larger than unity, which would be highly non-physical. From Eq. (8)–(10), we can now derive the following equation for the pressure evolution in the tissue.



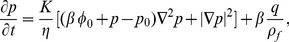
(11)A similar equation is frequently encountered in studies on flows in poro-elastic media [20], [21] and in pattern formation studies of air injection in granular media [22], where we here have assumed that both the relative variation of volume and the advective contribution from the tissue motion are negligible.

We shall here take the tip of the needle as our center of coordinates. For simplicity, the tissue surrounding the needle tip is assumed to be homogeneous and isotropic and the injected fluid will therefore be distributed in the tissue in a spherical symmetric way. The equation for the pressure evolution is most conveniently solved in spherical coordinates where the angular components by symmetry can be disregarded. Since the source term is located at the tip of the needle, we remove this term from the equation by considering the pressure evolution outside a small sphere surrounding the tip. The radius of this sphere is taken to be equal to the radius of the needle 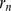 and is therefore assumed to be infinitesimal relative to the scales on which the fluid are distributed. From Eq. (8) we then get from Gauss theorem, and from the fact that mass cannot accumulate in an infinitesimal volume, a boundary condition on the form,
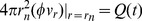
(12)where 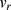 is the radial component of the fluid velocity and the flow rate 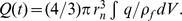 If we now make use of Darcy's law, we end with the following boundary condition for the pressure
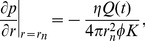
(13)where the porosity 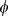 is calculated from the pressure through Eq. (10).

### Experimental data on subcutaneous injection

**Figure 6:**
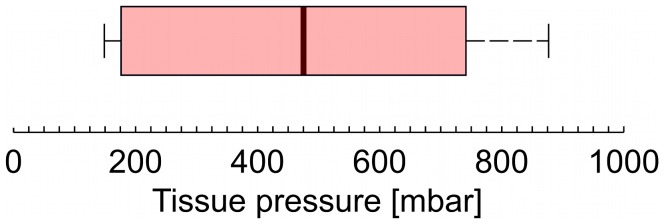
Average tissue counter pressure for eight patients. The boarder of the box is from the first to the third quartiles, with the median value marked as a black line. The whisker extend to the extreme values. The pressure is estimated from Eq. (4) using 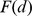 estimated from the reference measurements in air and the flow rate 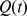 in tissue.

**Figure 7:**
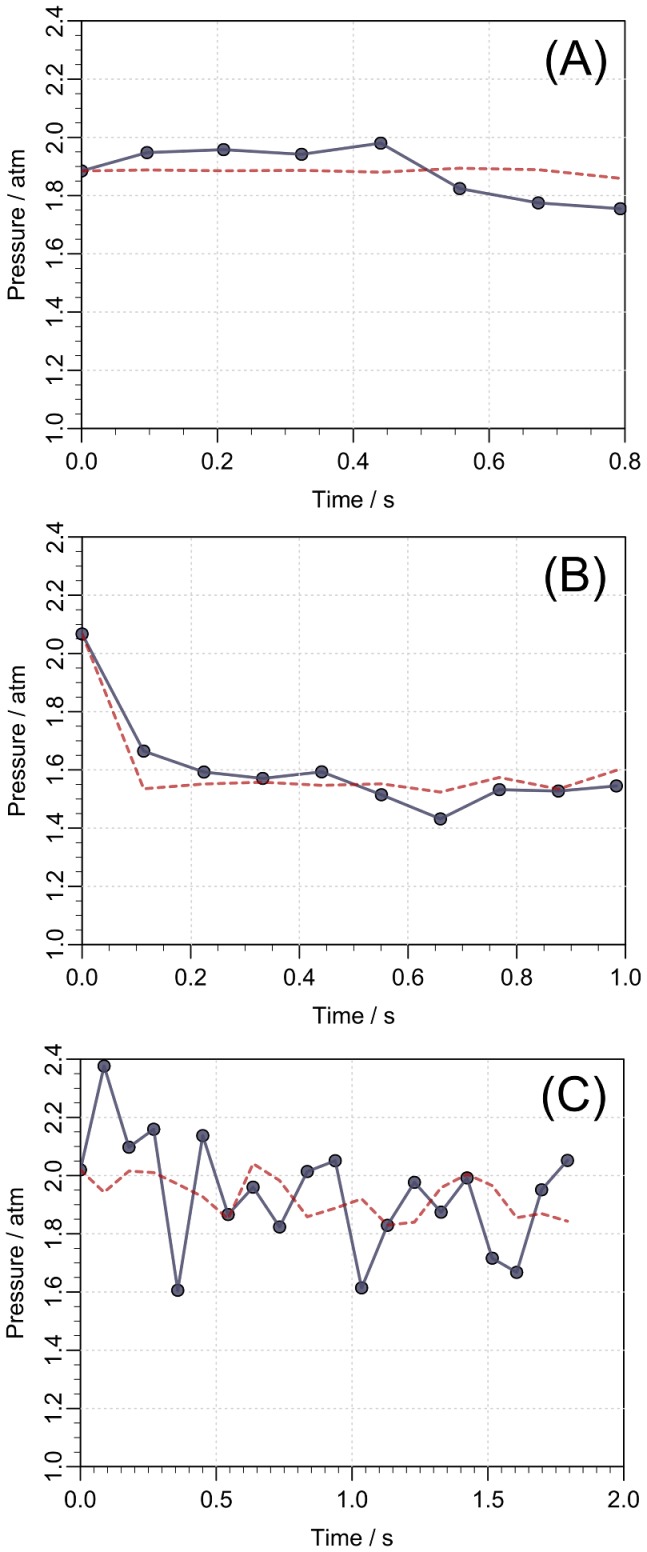
Pressure in the tissue during drug injection for three different patients. The pressure at the outlet of the needle (full line) is estimated from the spring force on the piston using the data on injection in air in Figure 5. The model prediction (dashed line) of the same pressure is computed from Eq. (11) and (12) by using the measured flow rate 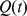 as a boundary condition. The pressure curves (A–C) corresponds to the flow rates shown in Figure 5 (A–C).

**Figure 8:**
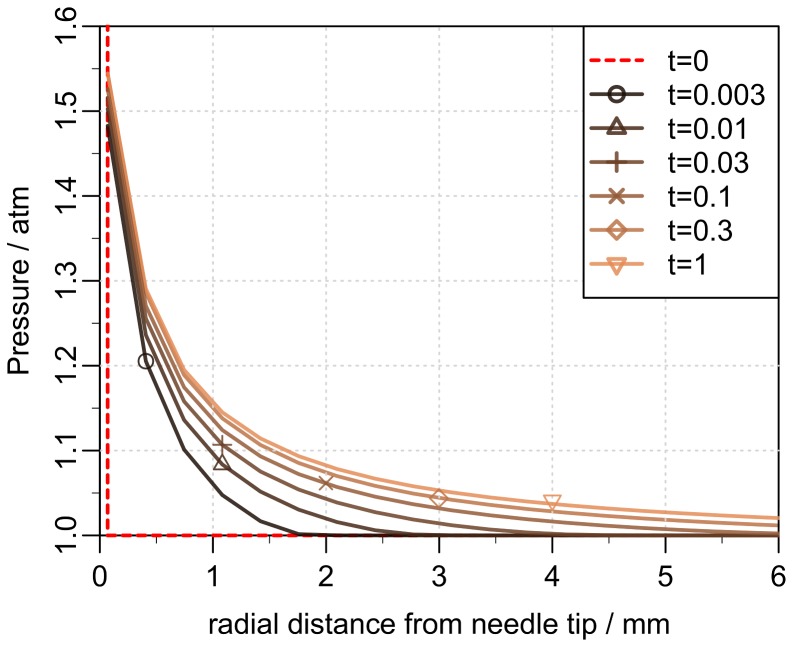
Pressure evolution in the tissue. The pressure in the tissue as function of the radial distance from the needle tip at different times (in seconds). Initially the over-pressure is localized around the tip of the needle and then quickly becomes distributed in the surrounding tissue.

As examples of the flow data, Figure 5 shows the measurements from 3 of the 11 patients. From Eq. (4), we can directly estimate the function 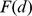 from the injections in air, since the atmospheric pressure, 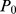, is known (note that variations in the atmospheric pressure have a negligible impact on our calculations and it is assumed that the friction force between the rubber piston and the syringe is independent on the flow rate). From our estimate of 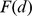, the drug injection rate and from Eq. (11) and (12), we can calculate the pressure evolution in the tissue once we know the model parameters 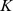, 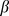 and 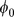. The other parameters like the viscosity of water (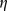) and atmospheric pressure (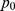) are known. The far field pressure in the tissue (away from the needle) is assumed to be equal to atmospheric pressure. In general, however, the pressure in subcutaneous tissue might be slightly below atmospheric pressure [13]. The pressure at the outlet of the needle tip is calculated from the function 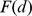 and the injection rate by using Eq. 4, where 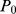 now is the pressure in the tissue and is a function of time. The average tissue counter pressure is shown in a box plot in Figure 6. Three patients has been omitted, as no change in the flow rate was observed indicating a low tissue resistance. We then compare the pressure 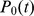 in the tissue with that predicted by the model at the needle tip, 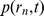, in order to find the free model parameters through a best fit (least squares). Note that in our calculation of 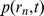, we use the flow rate as a boundary condition, given by Eq. (12). In Figure 7 we show 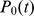 together with the model estimate 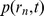 achieved by a best fit. The pressure evolution in radial distance from the needle tip and over time is presented in Figure 8. We see that the over-pressure is more or less localized in a sphere with a radius less than 5 mm, which is consistent with the distribution shown in Figure 3 (right).

**Figure 9:**
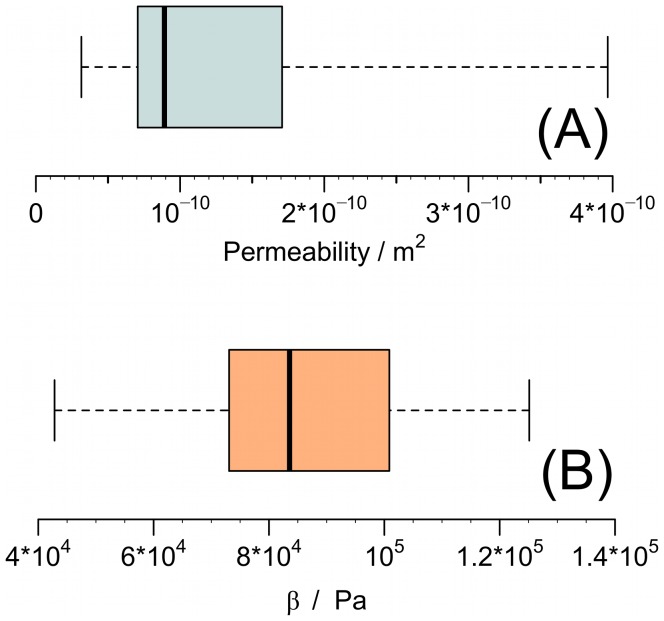
Box plots of the tissue permeability and bulk modulus. The values are estimated from best fits to the patient data. The permeability estimates are presented in panel (A) and the bulk modulus in panel (B). The middle line of the plot represents the value of the median and the outer edges of the box represent lower and upper quartiles, respectively. The whiskers extend to the extreme values.

In Figure 9A we show a box plot of the permeability computed from a best fit with the model to the patient data, again where three patients have been omitted. The typical permeability is from the model estimated to be of the order 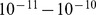 m^2^. From the interquartile range, we estimate the 95% confidence interval (the notch [23]) to be between 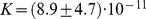 m^2^. Measurements of the permeability in porcine adipose tissue [24] gave values of the same order of magnitudes as reported in this article. The estimated values of the bulk modulus are shown in Figure 9B with a 95% confidence interval between the values 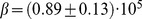 Pa. Note that at the outlet of the needle, the pressure in the tissue does for a few patients reach levels which are not fully consistent with a constitutive equation on the form of Eq. (10), i.e. the tissue porosity reaches values close to unity or might slightly exceed it. One way to remedy this is to change the constitutive equation and include higher order elastic or plastic effects. That being said, the pressure in the tissue does immediately drop slightly away from the needle tip (see Figure 8) and Eq. (10) becomes a valid description. Finally the background porosity 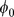 is in general very small and of the order 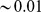.

## Discussion

Using a simple experimental setup we have been able to estimate the average tissue counter pressure during a subcutaneous injection with an insulin pen. The major advantages of this setup it that we do not modify the injection device and therefore are able to evaluate the pressure built up under normal injection conditions. We observe large variations in the counter pressure from a very low pressure, not detectable with this method, and up to pressures of about 800 mbar. All injections where performed with out complications, meaning that no skin reactions or pain during or after the injections were observed.

We have derived a model for the pressure evolution during injection in subcutaneous tissue, based on mass continuity as well as the basic laws of viscous flow in a poro-elastic medium. From application of the model to data on the insulin injections in diabetic patients, we have been able to determine flow permeability and bulk modulus of the tissue. Our model makes it possible to estimate how changes in the flow permeability and bulk modulus effect the pressure build-up in subcutaneous tissue during drug infusion, which is an important part of designing injection devices for insulin treatment. Control ensures that the device can deliver the full dose, that the back-flow through the injection channel is minimized and that injection pain or even tissue damage is reduced. Furthermore, our model, using the fitted parameters, is useful in general predictions of tissue pressure changes when mechanics of the injection device or the size of the needle is changed. Using the non-invasive method presented here the changes in counter pressure can easily be evaluated during clinical trails.

## Supporting Information

Supporting Information S1 Total pressure drop along the needle.
(PDF)
