# Supplementary material for: Model Study of the Pressure Build-Up during Subcutaneous Injection
Source: PLoS One. 2014 Aug 14;9(8):e104054. doi: 10.1371/journal.pone.0104054 (PMC4133188; doi:10.1371/journal.pone.0104054)
Supplement: Supporting Information S1 — Total pressure drop along the needle. (PDF) [file pone.0104054.s001.pdf]

Supporting Information: Total pressure drop along the needle

Entrance Flow in the Needle

We shall now consider the flow in the needle. The Reynolds number for the characteristic flow rates achieved during injection can be expressed in terms of the inner needle diameter D, the flow rate Q and the kinematic viscosity ν,

$$Re = \frac{4Q}{\pi D \nu}, \tag{1}$$

For a flow rate of approximately 200μL/s and a needle diameter of 135μm, we obtain $Re \sim 1900$. Even though this value is below the critical value for onset of turbulent flow in a pipe, we see that the inertial forces are not negligible. The flow in the entrance region of the pipe therefore differs from the laminar Poiseuille flow.

Due to the friction with the wall, boundary layers with increasing widths downstream will be formed at small distances from the needle inlet. In the boundary layers, the fluid particles experience a reduction of their velocity and owing to mass conservation the velocity of the particles near the axis must increase. In the entrance region the velocity field will vary not only in the radial direction but also in the axial direction[1]. However, sufficiently far from the inlet where the width of the boundary layer is approximately equal to the needle radius, the viscous forces will dominate and the velocity profile will become asymptotically parabolic as in the Poiseuille flow. The length of the entrance region when the boundary layers merge into each other is called the entry length, and we shall denote it by L_e . Since the inertial forces are not negligible in the entrance region a higher pressure gradient is needed to establish a given flow rate than in the case of a Poiseulle flow.

The development of flow patterns in the inlet of a 2D channel with parallel walls was studied in [1] and [2]. Details on the flow in the entrance of a pipe can be found in [3].

We shall here follow the calculations of [3] in order to calculate the pressure drop along the needle.

Governing equations

For this axially symmetrical flow the mass conservation equation, the radial and longitudinal Navier-Stokes equations read respectively,

$$\partial_z v_z + \frac{1}{r}\partial_r (r v_r) = 0, \tag{2}$$

$$v_z \partial_z v_r + v_r \partial_r v_r = -\frac{1}{\rho}\partial_r p + \frac{\nu}{r}\partial_r \left(\frac{1}{r}\partial_r (r v_r)\right) + \nu \partial_z^2 v_r \tag{3}$$

and

$$v_z \partial_z v_z + v_r \partial_r v_z = -\frac{1}{\rho}\partial_z p + \frac{\nu}{r}\partial_r \left(r \partial_r v_z\right) + \nu \partial_z^2 v_z. \tag{4}$$

The boundary conditions are,

$$v_z = U_o \qquad v_r = 0 \qquad z = 0, \forall_{(0 \le r \le R)}. \tag{5}$$

$$v_z = 2U_o \left(1 - \left(\frac{r}{R}\right)^2\right) \qquad v_r = 0 \qquad z = \infty, \forall_{(0 \le r \le R)}. \tag{6}$$

[1] This dependence of the velocity field on the axial coordinate constitutes a difference to Poiseuille flow in which for a given distance to the wall the velocity remains constant along the axial direction

$$v_z = v_r = 0 \qquad r = R, \forall_{(0<z<\infty)}. \tag{7}$$

We shall now simplify the Navier-Stokes equations given the relatively high Reynolds numbers estimated for the injection problem. The longitudinal velocity in the bulk of the flow is U_o. The boundary layer thickness, δ, is proportional to the square root of the kinematic viscosity of the fluid and it can be rigorously proven that near the inlet (see [1])

$$\frac{\delta(z)}{L} \sim \sqrt{\frac{\nu}{U_o z}}, \tag{8}$$

in which L denote a characteristic length of the variation of the axial velocity along the z coordinate, and $\delta(z)$ denote the boundary layer thickness at position z. The variation of the longitudinal velocity v_z takes place over distances equal to the entry length, in which $\delta(L_e) = R$ leading to the estimate[2]

$$\frac{R}{L_e} \sim \frac{1}{Re} \tag{9}$$

With the above estimate we conclude that

$$\frac{\partial_z^2 v_z}{\partial_r^2 v_z} \sim \frac{1}{Re^2}. \tag{10}$$

From the continuity equation we can see that $v_r \sim \frac{U_o}{Re}$. Similarly, making the estimation for the pressure variation in the entrance region from Eq. (4) we have

$$\Delta_z P \sim \rho U_o^2, \tag{11}$$

whereas from Eq. (3) we have for the transverse variation

$$\Delta_r P \sim \rho U_o^2 \frac{1}{Re^2} \tag{12}$$

Thus, we can consider that for high Reynolds number the pressure in a cross-section of the pipe is practically constant when compared with the z-variation. Then, for the typical Reynolds numbers of the injection we can describe the flow inside the needle by the following systems of symplified Navier-Stokes equations, known as Prandtl's boundary layer equations

$$\partial_z v_z + \frac{1}{r}\partial_r(r v_r) = 0, \tag{13}$$

$$v_z \partial_z v_z + v_r \partial_r v_z = -\frac{1}{\rho}\partial_z p + \frac{\nu}{r}\partial_r\left(r \partial_r v_z\right). \tag{14}$$

The solution of this system of equations was solved in [3]. There, the axial velocity was determined as

$$v_z = U_o \frac{I_o(\beta) - I_o(\beta \frac{r}{R})}{I_2(\beta)}, \tag{15}$$

in which I_n is the hyperbolic Bessel function of n'th order and $\beta = \beta(z)$ is a function of the z coordinate which can be determined as [3].

$$\frac{dz}{d\beta} = -Re \frac{R}{2} \frac{f'(\beta)}{g(\beta)} \qquad \beta \to \infty (z \to 0) \qquad \beta \to 0 (z \to \infty) \tag{16}$$

[2] A more accurate estimate was obtained in [3], see below

The functions $f(\beta)$ and $g(\beta)$ are determined by [3]

$$f(\beta) = \frac{4I_oI_2 - (I_o-1)^2 - 2I_1^2}{2I_2^2} \qquad g(\beta) = \beta^2 \frac{I_0 - 1 - I_2}{I_2} \tag{17}$$

The entry length is defined as the point where the central velocity has reached 99 of its terminal value. From the expression for the axial velocity the value of β at the entry length is $\beta_o \approx 0.69$. Thus, from Eq. (16) the ratio between the entry length and the pipe diameter is determined by

$$\frac{L_e}{D} \approx 0.057 \frac{U_o D}{\nu} \tag{18}$$

Pressure drop along the needle

Having the solution for the axial velocity and the estimation of the entry length, let us now calculate the pressure drop along the needle. Our starting point is the Prandtl equation for the longitudinal velocity

$$v_z \partial_z v_z + v_r \partial_r v_z = -\frac{1}{\rho}\frac{dp}{dz} + \frac{\nu}{r}\partial_r \left(r \partial_r v_z\right). \tag{19}$$

Evaluating the latter equation in $r = 0$, leads to

$$\left[\partial_z \frac{v_z^2}{2}\right]_{r=0} = -\frac{1}{\rho}\frac{dp}{dz} + \left[\frac{\nu}{r}\partial_r \left(r\partial_r v_z\right)\right]_{r=0}, \tag{20}$$

where we have used the fact that the pressure gradient is constant along the cross-section of the pipe and that both v_r and $\partial_r v_z$ are identically zero at the pipe axis. In order to calculate the pressure drop form the inlet to the entrance length L_e let us integrate Eq. (20) in the interval $z \in [0; L_e]$. Integration yields

$$\left[\frac{v_z^2}{2}\right]_{r=0,z=L_e} - \left[\frac{v_z^2}{2}\right]_{r=0,z=0} = -\frac{1}{\rho}(P(L_e) - P(0)) + \nu \int_0^{L_e} \left[\frac{1}{r}\partial_r \left(r\partial_r v_z\right)\right]_{r=0} dz, \tag{21}$$

Using the boundary conditions given by Eq. (5) and (6) we obtain

$$P(0) - P(L_e) = \Delta P_{L_e} = \frac{3}{2}\rho U_o^2 + \rho\nu \int_0^{L_e} \left[\frac{1}{r}\partial_r \left(r\partial_r v_z\right)\right]_{r=0} dz. \tag{22}$$

The first term in the right hand side of the Eq. (22) is essentially the pressure drop due to the acceleration of the fluid in the entrance region outside the boundary layer, while the second one accounts for the pressure losses owing to friction. In order to calculate the integral in Eq. (22) is more convenient to perform the integration changing, with the help of the definition in Eq. (16), the variable z to β.

$$\rho\nu \int_0^{L_e} \left[\frac{1}{r}\partial_r \left(r\partial_r v_z\right)\right]_{r=0} dz = -\rho\nu \int_{\beta_o}^{\infty} \left[\frac{1}{r}\partial_r \left(r\partial_r v_z\right)\right]_{r=0} \frac{dz}{d\beta} d\beta \approx 1.33 \rho U_o^2. \tag{23}$$

Pressure losses in the entrance region can be calculated accordingly,

$$\Delta P_{L_e} = \left(\frac{3}{2} + 1.33\right)\rho U_o^2. \tag{24}$$

Let us calculate now the pressure drop from the entry length to the outlet of the needle. In this region we have a fully developed parabolic profile, and the pressure difference can be determined by

$$P(L_e) - P(L) = \Delta P_E = \rho\nu \int_{L_e}^{L} \left[\frac{1}{r}\partial_r \left(r\partial_r v_z\right)\right]_{r=0} dz \tag{25}$$

Since we are in the region of the fully developed flow the integrand can be calculated formally taking the limit

$$\left[\frac{1}{r}\partial_r\left(r\partial_r v_z\right)\right]_{r=0} = \lim_{\beta\to 0}\left[\frac{1}{r}\partial_r\left(r\partial_r v_z\right)\right]_{r=0} \tag{26}$$

We thus obtain

$$\Delta P_E = \frac{8\rho\nu U_o}{R^2}(L - L_e) \tag{27}$$

Substituting in the above equation the expression given by Eq. (18) for L_e yields

$$\Delta P_E = \frac{128\eta L}{\pi D^4}Q - 1.82\rho U_o^2 \tag{28}$$

The total pressure losses in the needle can be obtained summing the pressure differences

$$\Delta P = \Delta P_{L_e} + \Delta P_E \tag{29}$$

From Eqs. (28) and (24) we can derive the expression for the total pressure drop in the needle as

$$\Delta P = \frac{128\eta L}{\pi D^4}Q(t) + \frac{16.16\rho}{\pi^2 D^4}Q^2(t). \tag{30}$$

References

1. Schlichting H, Gersten K (1979) Boundary-layer theory. McGraw-Hill.
2. Dyke MV (1970) Entry flow in a channel. Journal of Fluid Mechanics 44: 813–823.
3. Lautrup B (2011) Physics of Continuous Matter. Second Edition: Taylor & Francis.
